# Response to pioglitazone in non-alcoholic fatty liver disease patients with *vs*. without type 2 diabetes: A meta-analysis of randomized controlled trials

**DOI:** 10.3389/fendo.2023.1111430

**Published:** 2023-03-29

**Authors:** Zeyu Wang, Huiqing Du, Ying Zhao, Yadi Ren, Cuihua Ma, Hongyu Chen, Man Li, Jiageng Tian, Caihong Xue, Guangfeng Long, Meidong Xu, Yong Jiang

**Affiliations:** ^1^ Endoscopy Center, Department of Gastroenterology, Shanghai East Hospital, School of Medicine, Tongji University, Shanghai, China; ^2^ Department of Gastroenterology, Xingtai People’s Hospital, Xingtai, China; ^3^ Department of Gastroenterology, The Second Hospital of Tianjin Medical University, Tianjin, China; ^4^ Department of Gastroenterology, The Second Affiliated Hospital of Baotou Medical College, Inner Mongolia University of Science and Technology, Baotou, China; ^5^ Department of Pediatric Ophthalmology and Strabismus, Tianjin Eye Hospital, Tianjin, China; ^6^ Department of Clinical Laboratory, Children’s Hospital of Nanjing Medical University, Nanjing, China

**Keywords:** pioglitazone, nonalcoholic fatty liver disease, randomized controlled trials, diabetes mellitus, nonalcoholic steatohepatitis

## Abstract

**Background:**

Pioglitazone is considered a potential therapy for non-alcoholic fatty liver disease (NAFLD). However, different effects of pioglitazone on NAFLD have been demonstrated in diabetic and non-diabetic patients. Herein, a meta-analysis of randomized, placebo-controlled trials was carried out to indirectly compare pioglitazone in NAFLD patients with *vs*. without type 2 diabetes.

**Methods:**

Randomized controlled trials (RCTs) of pioglitazone *vs*. placebo involving NAFLD patients with or without type 2 diabetes/prediabetes collected from databases were enrolled into this analysis. Methodological quality was employed to evaluate the domains recommended by the Cochrane Collaboration. The analysis covered the changes in histology (fibrosis, hepatocellular ballooning, inflammation, steatosis), liver enzymes, blood lipids, fasting blood glucose (FBS), homeostasis model assessment-IR (HOMA-IR), weight and body mass index (BMI) before and after treatment, and adverse events.

**Results:**

The review covered seven articles, with 614 patients in total, of which three were non-diabetic RCTs. No difference was found in patients with *vs*. without type 2 diabetes in histology, liver enzymes, blood lipids, HOMA-IR, weight, BMI, and FBS. Moreover, no significant difference was revealed in adverse effects between NAFLD patients with diabetes and without DM, except the incidence of edema that was found to be higher in the pioglitazone group than in the placebo group in NAFLD patients with diabetes.

**Conclusions:**

Pioglitazone could exert a certain effect on alleviating NAFLD, which was consistent between non-diabetic NAFLD patients and diabetic NAFLD patients in improving histopathology, liver enzymes, and HOMA-IR and reducing blood lipids. Furthermore, there were no adverse effects, except the incidence of edema which is higher in the pioglitazone group in NAFLD patients with diabetes. However, large sample sizes and well-designed RCTs are required to further confirm these conclusions.

## Introduction

The overall prevalence of non-alcoholic fatty liver disease (NAFLD) is globally estimated at 25%–40%, which has been considered a major disease burden worldwide with a rising trend (1). Non-alcoholic steatohepatitis (NASH) will be developed in approximately 25% of NAFLD patients, of whom one-fourth will develop liver failure and hepatocellular carcinoma (HCC) with higher rates of progression to cirrhosis (2–4). Indeed, a study in the US has already demonstrated that NAFLD is the most common risk factor for HCC (24%), in contrast to HCV (23%) and hepatitis B (19.3%) (5). NAFLD could exhibit a close correlation with metabolic syndrome, a range of risk factors for type 2 diabetes mellitus, and end-stage vascular disease, with cardiovascular disease being the most common burden of death in patients with NAFLD (6). Lifestyle interventions, such as calorie restriction and exercise therapy, are demonstrated to play a central role in treating NAFLD, which, however, are difficult to achieve and maintain. Despite several pharmacologic interventions to treat NAFLD, there is still no approved drug for its effective treatment (3, 7).

Pioglitazone as a peroxisome proliferator-activated receptor (PPAR) agonist could increase plasma adiponectin levels, which are associated with insulin sensitivity improvement, exerting anti-inflammatory and antifibrotic effects on NAFLD (8). Della et al. discovered that treatment with pioglitazone at low dosage significantly improved liver inflammation and alleviated insulin resistance in NAFLD patients with type 2 diabetes mellitus (T2DM) (9). Bril et al. found that pioglitazone discontinuation in patients with biopsy-proven NASH was associated with biochemical worsening of the disease, and pioglitazone therapy in patients with NASH should be considered as a long-term treatment (10). These studies suggest that pioglitazone has a certain role in the treatment of NAFLD. As a result, pioglitazone may be recommended for treating NAFLD as verified by the improvement of liver histology and some biochemical indexes in several studies (11–15). These studies have explored the efficacy of pioglitazone in NAFLD patients, primarily by comparing the effect of pioglitazone and all other drugs for NAFLD together. Furthermore, these studies have not compared NAFLD patients with T2DM to non-diabetic patients, and there are varying opinions among these studies. For this reason, it is of significance to investigate whether pioglitazone will exert different effects between diabetic and non-diabetic individuals, so as to treat different types of NAFLD more efficiently.

This meta-analysis was carried out to compare the efficacy and safety of pioglitazone in treating NAFLD with *vs*. without T2DM. Nevertheless, few studies have compared pioglitazone with placebo in patients with NAFLD between T2DM and normal glucose tolerance; therefore, we conducted this study to try to replenish this gap.

## Materials and methods

### Retrieval strategy

**Figure 1:**
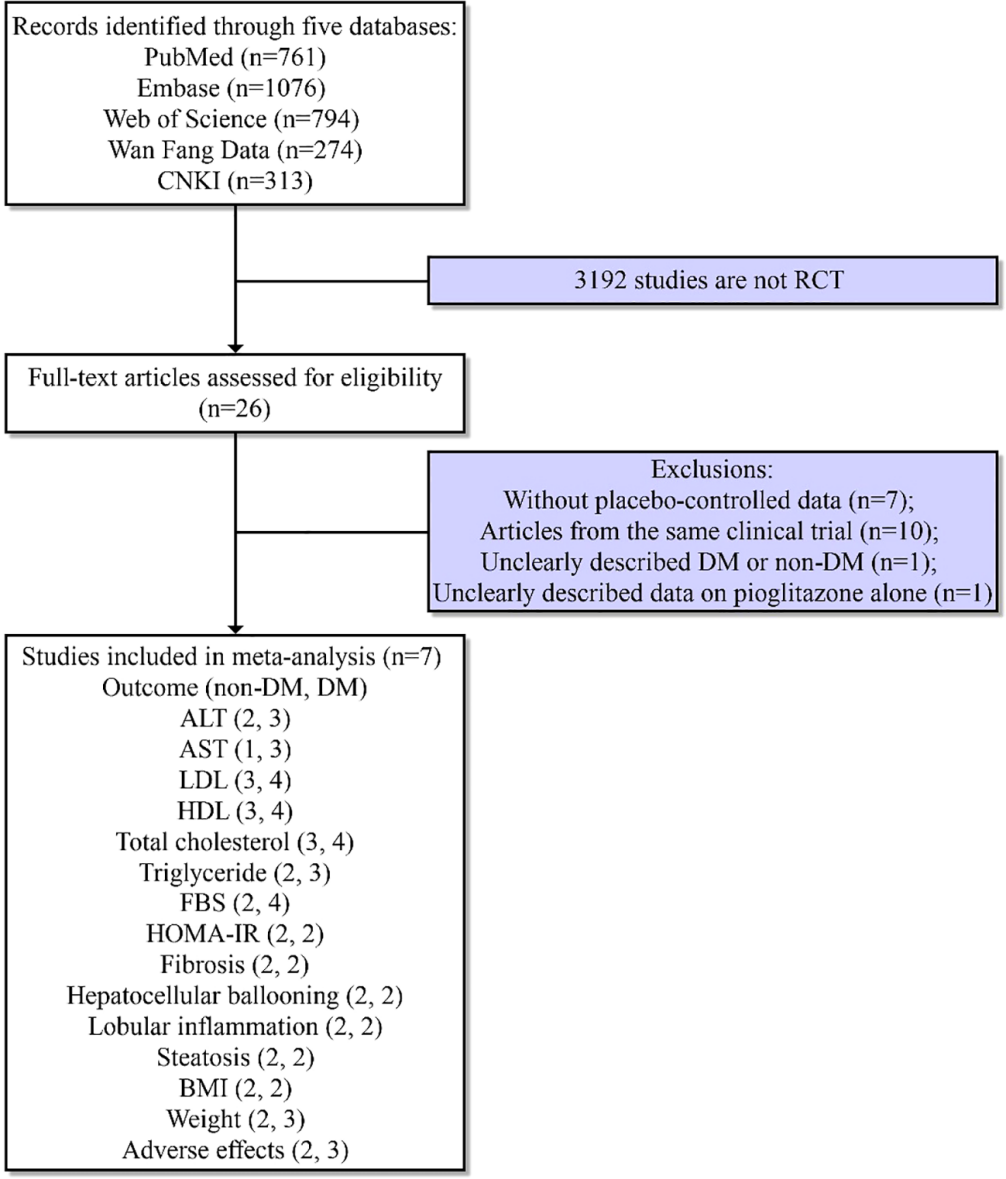
Flowchart of study information.

The major databases PubMed, Embase, Web of Science, WangFang Data, CNKI, and Medline were systematically searched for literature to retrieve eligible studies without language restriction by two reviewers from inception to May 2022, and additional information or raw data were asked by the corresponding authors through email. The keywords “nonalcoholic steatohepatitis” OR “nonalcoholic fatty liver disease” OR “NASH” OR “NAFLD” AND “pioglitazone” were employed. At the same time, a wide scanning of relevant references listed in the retrieved articles was also conducted to seek other articles of possible eligibility. The research selection process is provided in **Figure 1**.

### Inclusion and exclusion criteria

Randomized controlled trials of pioglitazone *vs*. placebo involving patients with NAFLD confirmed by liver biopsy or ultrasound, with or without T2DM/prediabetes, were included. The exclusion criteria were as follows: i) non-randomized placebo-controlled trials; ii) trials without raw data; iii) leading articles, abstracts, letters, animal experiments, case reports, meta-analysis, expert opinion, conference papers, and book sections; iv) no clear validity of whether NAFLD patients were complicated with diabetes; v) patients with severe renal failure, heart failure, malignant tumor, or secondary hepatic fat accumulation such as viral hepatitis or significant alcohol consumption; and vi) trials that did not present data on pioglitazone alone.

### Methodological quality assessment

**Figure 2:**
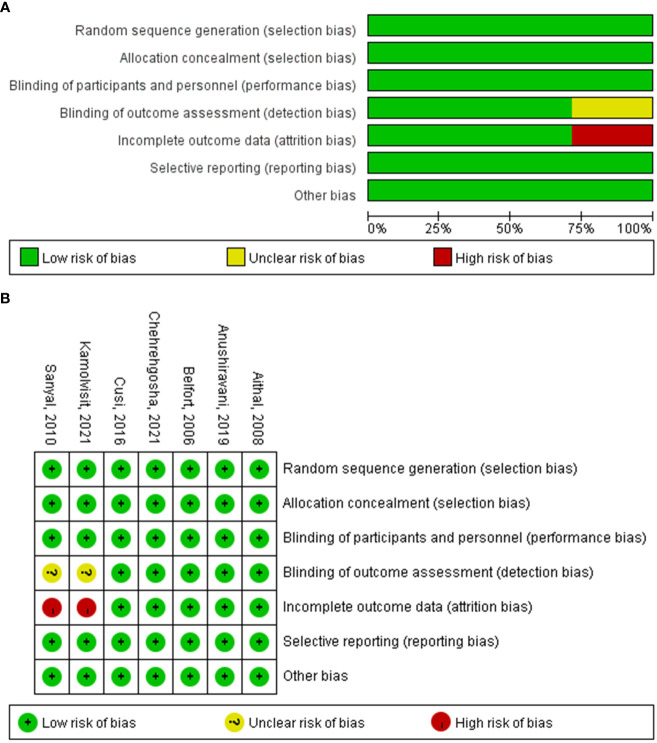
Methodological quality **(A)** and risk of bias **(B)** for trials included in systematic review.

Each randomized controlled trial was evaluated for methodological quality using Cochrane Collaboration’s tool (16), which involved sequence generation, allocation hiding, the blinding method in the selection of participants and personnel and result evaluators, processing of data results, and the lack of other deviation sources, determining the high, low, or unclear deviation risk of the research. The assessment of the enrolled studies is presented in **Figure 2**.

### Outcome measures

The primary outcomes referred to histological variables such as fibrosis, steatosis, inflammation, and hepatocellular ballooning, and the secondary outcomes included changes in alanine transaminase (ALT), aspartate aminotransferase (AST), FBS, blood lipids, HOMA-IR, weight, and BMI. In addition, the impact on adverse events was evaluated.

### Data extraction

Data were extracted by two reviewers independently and summarized into a standardized spreadsheet in duplicate after the studies have been confirmed to meet the predetermined criteria. Disagreements were resolved by negotiated solutions or mutual discussion, and the quality of the trials was assessed by kappa statistics scoring. The following variables were extracted from each study: i) general information (name of the first author, year, study design, presence of diabetes); ii) treatment details (dosage, frequency, duration, lifestyle changes throughout the trial); iii) histological variables (baseline and at the end of the study): fibrosis, steatosis, inflammation, and hepatocellular ballooning; iv) laboratory and anthropometric tests (baseline and at the end of the study), covering ALT, AST, blood lipids, FBS, HOMA-IR, weight, and BMI; and v) adverse events.

### Data analysis

All data were analyzed on R software v3.6.1 (R Foundation for Statistical Computing, Vienna, Austria). The “Meta” package was employed in the meta-analysis. Mean differences were calculated by the following formula: (mean value of treatment at baseline − mean value of treatment at the end of the study) − (mean value of control at baseline − mean value of control at the end of the study). The mean differences for the intervention and control groups were either directly provided by the research results or calculated by the mean values before and after treatment. To calculate the SD of the change in means for those studies, it was imputed applying a modified method by Follmann et al.: SDchange in means = sq root [(SDpre)2 + (SDpost)2 − (2(q)·SDpre·SDpost)] (17). The change in means (SDchange in means) was obtained using the SD of the preintervention mean (SDpre) and the SD of the postintervention mean (SDpost) as well as the within-participant correlation (*q*) of the outcome measures. Sensitivity analysis was conducted to exclude studies that influence the stability of research results and to assess heterogeneity. Publication bias was evaluated with funnel plot analysis and Egger’s and Begg’s tests. The level of statistical significance was 0.05, and the statistical heterogeneity across studies was represented by *I* ^2^ statistics. Improvement was determined by a reduction of 1 point or more in the pathology score. The fixed-effects model will be employed in the statistical analysis when *P* ≥0.05 and *I* ^2^ ≤50%; otherwise, the random-effects model was applied. Dichotomous and continuous variables were expressed as odds ratios (ORs), mean differences (MDs), and 95% confidence intervals (CIs), respectively.

## Result

### Study characteristics

After the primary screening, 26 studies were included for the subsequent full-text review until May 2022. Seven articles (9, 18–23) without placebo-controlled data, 10 articles (24–33) from the same clinical trial, one article (34) without a clear statement of whether NAFLD patients were complicated with diabetes, and one article (35) that did not present data on pioglitazone alone were removed. Ultimately, a total of seven studies (11, 12, 36–40) deemed eligible were included, covering 614 patients, three of which (36–38) were non-diabetic RCTs, each being extracted for outcomes. The subjects of four studies included patients with NASH, and three studies included patients with NAFLD. The mean age of the patients with diabetes or prediabetes *vs*. without diabetes or prediabetes was found to be 51.1 ± 8.4 *vs*. 49.3 ± 11 years, and the male sex distribution was 59.3% *vs*. 49.1%. The main characteristics of the RCTs involved in the network meta-analysis are summarized in **Table 1** and **Supplementary Table 1**. The flowchart in **Figure 1** describes the selection process of the literature and the final selection of the studies. The dose of pioglitazone ranged from 15 to 45 mg/day, and the duration of pioglitazone or placebo treatment ranged from 3 to 24 months.

**Table 1:** Patient and trial characteristics of the included studies.

Study	*N*	Intervention, dose	Comparator(s)	Duration	Diabetes or prediabetes	NASH or NAFLD	Country	NASH/NAFLD assessment in results
Aithal (36),	74	Pioglitazone, 30 mg/day	Placebo	12 months	No	NASH	United Kingdom	Histology
Anushiravani (37),	60^a^	Lifestyle + pioglitazone, 15 mg/day	Lifestyle + placebo	3 months	No	NAFLD	Iran	Ultrasound
Sanyal (38),	163^a^	Pioglitazone, 30 mg/day	Placebo	24 months	No	NASH	America	Histology
Belfort (11),	47^a^	Hypocaloric diet + pioglitazone, 45 mg/day	Hypocaloric diet + placebo	6 months	Yes	NASH	America	Histology
Cusi (12),	101	Pioglitazone, 45 mg/day	Placebo	18 months	Yes	NASH	America	Histology
Kamolvisit (39),	98	Pioglitazone, 45 mg/day	Placebo	18 months	Yes	NAFLD	Thailand	Ultrasound
Chehrehgosha (40),	71^a^	Pioglitazone, 30 mg/day	Placebo	6 months	Yes	NAFLD	Iran	Ultrasound

a Represents patients in the trial arms of interest only.

### Study quality assessment

The risk of bias (such as selection bias, performance bias, detection bias, attrition bias, and reporting bias) was assessed using Cochrane Collaboration’s tool. All data were derived from randomized studies. The probability of bias was estimated and considered low in most studies and domains (**Figure 2**).

### Changes in liver histology with pioglitazone

**Figure 3:**
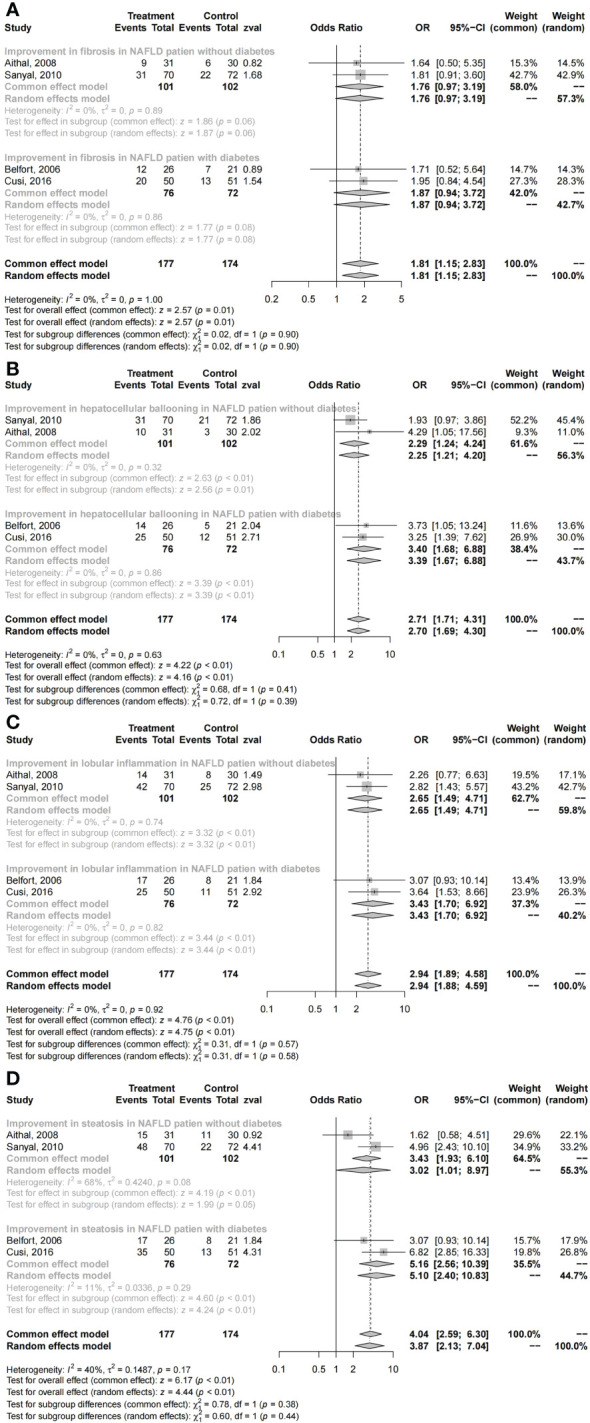
Changes in histology with pioglitazone: **(A)** fibrosis, **(B)** hepatocellular ballooning, **(C)** lobular inflammation, and **(D)** steatosis.

The histological changes of the liver were significantly improved in NAFLD patients who received pioglitazone therapy (fibrosis: *I* ^2^ = 0, OR = 1.81, 95% CI: 1.15 - 2.83, *P* = 0.01; hepatocellular ballooning: *I* ^2^ = 0, OR = 2.71, 95% CI: 1.71 - 4.31, *P* < 0.01; lobular inflammation: *I* ^2^ = 0, OR = 2.94, 95% CI: 1.89 - 4.59, *P* < 0.01; steatosis: *I* ^2^ = 40%, OR = 4.04, 95% CI: 2.59 - 6.30, *P* < 0.01; **Figure 3**). No significant differences in primary outcomes were found in NAFLD patients with diabetes compared with those without diabetes who received pioglitazone therapy (fibrosis: *χ* ^2^ = 0.02, *P* = 0.90; hepatocellular ballooning: *χ* ^2^ = 0.68, *P* = 0.41; lobular inflammation: *χ* ^2^ = 0.31, *P* = 0.57; steatosis: *χ* ^2^ = 0.78, *P* = 0.38; **Figure 3**).

The subgroup comparison results revealed no obvious superiority of pioglitazone therapy in fibrosis both in NAFLD patients with diabetes and without diabetes (with DM: OR = 1.87, 95% CI: 0.94 - 3.72, *P* = 0.08; without DM: OR = 1.76, 95% CI: 0.97 - 3.19, *P* = 0.06; **Figure 3**). However, these results suggest that pioglitazone may play a role in the treatment of liver fibrosis, with significant improvements in hepatocellular ballooning (with DM: OR = 3.40, 95% CI: 1.68 - 6.88, *P* < 0.01; without DM: OR = 2.29, 95% CI: 1.24 - 4.24, *P* < 0.01; **Figure 3**), lobular inflammation (with DM: OR = 3.43, 95% CI: 1.70 - 6.92, *P* < 0.01; without DM: OR = 2.65, 95% CI: 1.49 - 4.71, *P* < 0.01; **Figure 3**), and steatosis (with DM: OR = 5.16, 95% CI: 2.56 - 10.39, *P* < 0.01; without DM: OR = 3.02, 95% CI: 1.01 - 8.97, *P* = 0.05; **Figure 3**) compared with placebo.

### Changes in liver enzymes with pioglitazone

AST and ALT were confirmed to be significantly decreased in NAFLD patients who received pioglitazone therapy (AST: *I* ^2^ = 51%, MD = −6.56, 95% CI: (−11.18) - (−1.94), *P* < 0.01; ALT: *I* ^2^ = 71%, MD = −14, 95% CI: (−23.75) - (−4.26), *P* < 0.01; **Supplementary Figure 1**). No significant differences were found in both AST and ALT between NAFLD patients with diabetes and those without diabetes who received pioglitazone therapy (AST: *χ* ^2^ = 0.19, *P* = 0.66; ALT: *χ* ^2^ = 0.16, *P* = 0.69; **Supplementary Figure 1**).

The subgroup comparison indicated no significant improvements in both AST and ALT in NAFLD patients without diabetes who received pioglitazone therapy compared with those who received placebo [AST: MD = −5.5, 95% CI: (−11.33) - 0.33, *P* = 0.06; ALT: MD = −17.79, 95% CI: (−38.14) - 2.57, *P* = 0.09; **Supplementary Figure 1**], while there was a significant reduction in AST in patients with diabetes [MD = −7.48, 95% CI: (−14.27) - (−0.7), *P* = 0.03; **Supplementary Figure 1**], but not in ALT [MD = −12.74, 95% CI: (−26.33) - 0.84), *P* = 0.07; **Supplementary Figure 1**].

### Changes in metabolism with pioglitazone

**Figure 4:**
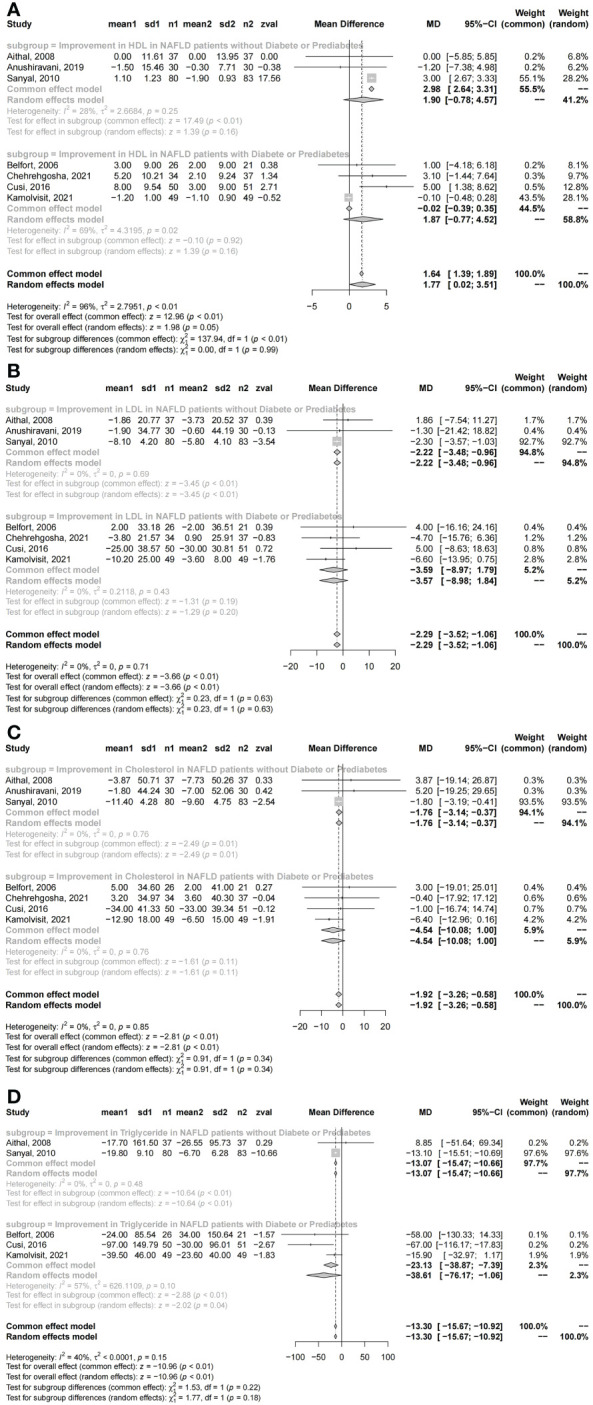
Changes in metabolism with pioglitazone: **(A)** HDL, **(B)** LDL, **(C)** total cholesterol, and **(D)** triglycerides.

HDL and HOMA-IR were confirmed to be significantly improved in NAFLD patients who received pioglitazone therapy; however, the levels of LDL, total cholesterol, triglycerides, and FBS showed no significant changes compared with the placebo groups. No significant differences were found in NAFLD patients with diabetes compared with those without diabetes who received pioglitazone therapy in terms of HDL, LDL, total cholesterol, triglycerides, HOMA-IR, and FBS (HDL: *I* ^2^ = 96%, *χ* ^2^ = 0.00, *P* = 0.99; LDL: *I* ^2^ = 0%, *χ* ^2^ = 0.23, *P* = 0.63; total cholesterol: *I* ^2^ = 0%, *χ* ^2^ = 0.91, *P* = 0.34; triglycerides: *I* ^2^ = 40%, *χ* ^2^ = 1.53, *P* = 0.22; HOMA-IR: *I* ^2^ = 92%, *χ* ^2^ = 1.30, *P* = 0.25; FBS: *I* ^2^ = 81%, *χ* ^2^ = 2.42, *P* = 0.12; **Figure 4** and **Supplementary Figure 2**).

The subgroup comparison results showed significant improvements in HDL, LDL, total cholesterol, and triglycerides with pioglitazone therapy than with placebo in patients without diabetes [HDL: MD = 2.98, 95% CI: 2.64 - 3.31, *P* < 0.01; LDL: MD = −2.22, 95% CI: (−3.48) - (−0.96), *P* < 0.01; total cholesterol: MD = −1.76, 95% CI: (−3.14) - (−0.37), *P* = 0.01; triglycerides: MD = −13.07, 95% CI: (−15.47) - (−10.66), *P* < 0.01; **Figure 4**], while there were no significant improvements in both FBS and HOMA-IR [FBS: MD = −6.16, 95% CI: (−22.14) - 9.81, *P* = 0.45; HOMA-IR: MD = −0.43, 95% CI: (−2.06) - 1.2, *P* = 0.60; **Supplementary Figure 2**]. However, no significant improvements were found in NAFLD patients with diabetes in HDL, LDL, and total cholesterol [HDL: MD = 1.87, 95% CI: (−0.77) - 4.52, *P* = 0.16; LDL: MD = −3.59, 95% CI: (−8.97) - 1.79, *P* = 0.19; total cholesterol: MD = −4.54, 95% CI: (−10.08) - 1.00, *P* = 0.11; **Figure 4**]. Significant improvements were revealed in triglycerides, FBS, and HOMA-IR in NAFLD patients with diabetes [triglycerides: MD = −38.61, 95% CI: (−76.17) - (−1.06), *P* = 0.04; FBS: MD = −21.84, 95% CI: (−23.06) - (−20.63), *P* < 0.01; HOMA-IR: MD = −1.82, 95% CI: (−3.57) - (−0.07), *P* = 0.04; **Figure 4** and **Supplementary Figure 2**].

### Changes in weight and BMI with pioglitazone

Weight and BMI showed no significant differences in patients who received pioglitazone therapy and those who received a placebo. No significant differences were found in both weight and BMI between NAFLD patients with diabetes and those without diabetes who received pioglitazone therapy (weight: *I* ^2^ = 0%, *χ* ^2^ = 1.15, *P* = 0.28; BMI: *I* ^2^ = 0%, *χ* ^2^ = 0.07, *P* = 0.79; **Supplementary Figure 3**).

The subgroup comparison results revealed significant increases in both weight and BMI compared with the placebo groups in patients without diabetes (weight: MD = 4.15, 95% CI: 2.14 - 6.17, *P* < 0.01; BMI: MD = 0.84, 95% CI: 0.03 - 1.65, *P* = 0.04; **Supplementary Figure 3**). No significant difference in BMI [MD = 0.64, 95% CI: (−0.58) - 1.87, *P* = 0.30] or weight [MD = 1.77, 95% CI: (−2.09) - 5.63, *P* = 0.37; **Supplementary Figure 3**] was found in NAFLD patients with diabetes.

### Adverse effects of pioglitazone compared with placebo

No significant differences were revealed in terms of adverse effects between NAFLD patients with diabetes and those without diabetes who received pioglitazone therapy (*I* ^2^ = 61%, *χ* ^2^ = 3.44, *P* = 0.06; **Supplementary Figure 4**).

No significant difference was found in terms of adverse effects between pioglitazone and placebo in NAFLD patients with or without diabetes. The mean differences and 95% CI for patients with diabetes and without diabetes with NAFLD were calculated as follows: DM: OR = 1.61, 95% CI: 0.82 - 3.16, *P* = 0.17; without DM: OR = 0.47, 95% CI: 0.16 - 1.42, *P* = 0.18 (**Supplementary Figure 4**). The incidence of edema was significantly increased in the pioglitazone group than in the placebo group in NAFLD patients with DM. No statistical significance was found in specific adverse effects comparing the pioglitazone group with the corresponding placebo group (**Table 2**).

**Table 2:** Reported adverse events and withdrawals during the treatment period.

Adverse Events	NAFLD with DM			NAFLD without DM		
	Placebo (*n* = 141)	Pioglitazone (*n* = 152)	*P*	Placebo (*n* = 120)	Pioglitazone (*n* = 111)	*P*
Cardiovascular	9	4	0.116	14	10	0.507
Gastrointestinal	17	14	0.429	7	4	0.423
Hypoglycemic	9	5	0.213	8	15	0.081
Neurologic	6	9	0.516	6	2	0.173
Gynecologic	2	2	0.94	0	1	0.225
Urologic	4	7	0.423	0	0	-
Edema	3	12	0.02	0	0	-
Musculoskeletal	21	23	0.955	4	4	0.911
Hepatotoxicity	1	0	0.226	6	4	0.601
Bone fractures	0	0	-	5	3	0.541
Cancer	1	0	0.226	0	0	-
Total number of withdrawals	3	2	0.591	7	6	0.888

### Sensitivity analysis and publication bias

We conducted a sensitivity analysis and publication bias analysis on the research studies with significant heterogeneity. Running the sensitivity analysis by excluding some high-risk studies showed a remarkable effect on the results of the analysis. Excluding the studies of Chehrehgosha et al. (40) in the ALT analysis, Anushiravani et al. (37) in the HDL analysis, Kamolvisit et al. (39) in the FBS analysis, and Cusi et al. (12) in HOMA-IR changes the substantiation of the corresponding results of the meta-analysis (**Supplementary Figure 5**). The analysis of the funnel plot for publication bias is shown in **Supplementary Figure 6**. Furthermore, Begg’s test showed no publication bias in ALT, FBS, HDL, and HOMA-IR analysis (all *P* > 0.05).

## Discussion

The present guidelines state the promising role of pioglitazone in liver histology in NASH patients as confirmed by liver biopsy, whether or not suffering from T2DM; however, the safety of long-term treatment should also be considered (41, 42). Tokushige et al. (43) recommend pioglitazone for NASH patients with insulin resistance. A prospective study (44) aiming at adults with biopsy-proven NASH (49 with prediabetes and 52 with T2DM) suggested pioglitazone for NASH patients with prediabetes as well as for NASH patients with T2DM to achieve metabolic and histologic benefits. However, this head-to-head observational study may lead to erroneous results with inconsistent baselines. Previous meta-analyses (13, 45–48) have explored the efficacy of pioglitazone in the treatment of NAFLD, primarily by comparing the effect of pioglitazone and all other drugs for NAFLD together and obtaining similar conclusions that pioglitazone has effects on NAFLD patients with T2DM or non-diabetes. Furthermore, studies have not compared NAFLD patients with T2DM to NAFLD patients without diabetes. As a result, no convincing conclusions about pioglitazone in the treatment of NAFLD patients without diabetes can be indeed drawn. In order to obtain a better understanding of the effects of pioglitazone in non-diabetes and diabetes NAFLD, RCTs on pioglitazone in the treatment of diabetes or non-diabetes NAFLD were searched and compared with placebo, so as to achieve an indirect comparison of pioglitazone in the treatment of NAFLD with *vs*. without diabetes, comprehending the efficacy and adverse effects of pioglitazone in the treatment of NAFLD patients.

The improvement of liver fibrosis is of crucial significance for the treatment of NAFLD as it is associated with higher rates of cirrhosis as well as overall mortality (1, 2). Mahady et al. (49) have stated that pioglitazone can optimize histological variables, such as fibrosis, hepatocellular ballooning, lobular inflammation, and steatosis. As Musso et al. (45) stated, pioglitazone can contribute to reversing advanced fibrosis in NASH, even in non-diabetic patients. However, the article has not compared the effects of non-diabetes NAFLD with diabetes NAFLD, but only compared pioglitazone with different drugs. We demonstrated the outcomes of pioglitazone in NAFLD patients on improvements in fibrosis, hepatocellular ballooning, lobular inflammation, and steatosis, which were similar to the results of the placebo group. The subgroup comparison results revealed the association of pioglitazone with significant improvements in hepatocellular ballooning, lobular inflammation, and steatosis both in NAFLD patients with diabetes and without diabetes compared with placebo. Though no significant improvements in fibrosis were found both in NAFLD patients with diabetes and without diabetes, it may be related to the relatively limited sample size, and both groups have trends of improvement.

Van et al. (50) reported that pioglitazone can improve liver biochemistry in mice deficient in phosphatidylethanolamine N-methyltransferase by activating PPARγ, which redirects the flux of fatty acids toward the adipose tissue away from the liver. Mahady et al. (49) concluded that thiazolidinediones can improve liver biochemistry by lowering ALT. In this review, we discovered the same effects of pioglitazone on improvements in both ALT and AST compared with diabetes NAFLD. The subgroup comparison results showed significant reductions in AST only in patients with diabetes (*P* = 0.003), while improvement was exhibited in the liver enzymes in both groups. The absence of statistical significance may be attributed to the high heterogeneity, limited sample size, and the calculated SD value.

The effect of pioglitazone on blood lipids varies among patients with NAFLD. Aithal et al. (36) confirmed the inhibitory role of pioglitazone in LDL but not in TC and HDL. Anushiravani et al. (37) concluded that pioglitazone can reduce LDL and TC. Pioglitazone can elevate plasma adiponectin levels, which is conducive to improving insulin sensitivity. We observed no significant differences in HDL, LDL, total cholesterol, triglycerides, FBS, and HOMA-IR between NAFLD patients with diabetes and those without diabetes who received pioglitazone therapy. Subgroup analysis showed a reduction of blood lipids to some extent in NAFLD patients with or without diabetes by pioglitazone. The higher baseline FBS values and greater room for improvement of patients with diabetes may affect the statistical results.

Pioglitazone serves as a prominent regulator of adipocyte differentiation and adipogenesis, which can lead to weight gain and obesity with chronic stimulation (51). Similar to previous results (42, 49, 51), in terms of variations in weight and BMI, we revealed significant differences in the two indexes between non-diabetes patients treated with pioglitazone and those with a placebo. However, an increase in weight can be found in NAFLD patients with diabetes, and the results showed no significant difference. The increase in weight caused by pioglitazone may be related to water–sodium retention and increased fat content (52, 53). These results still need to be studied with a larger sample size.

Drug safety is one of the key factors in the practicability of a drug. As a hypoglycemic drug, the application of pioglitazone in non-diabetes patients remains controversial. Some studies (54, 55) have suggested the contributed development of bladder cancer by the long-term use of pioglitazone, but others (56, 57) argued otherwise. A meta-analysis (58) revealed the increased risk of congestive heart failure by the use of glitazones, and another article (59) indicated its contribution to the increased risks of bone fracture. However, the patients involved in these articles are mainly diabetes patients, who require a long-term administration of pioglitazone, and the observed patients are the same. In the meta-analysis, pioglitazone could be well tolerated, and no major adverse events were found in the relevant literature. We noticed no significant adverse effects between NAFLD patients who received pioglitazone therapy and those who received a placebo. No statistical significance was found in the specific adverse effects of most groups compared with the corresponding placebo group, including cancer, congestive heart failure, and bone fracture. The incidence of edema was found to be higher in the pioglitazone group than in the placebo group in NAFLD patients with diabetes. Although pioglitazone has the risk of causing water and sodium retention (60), however, the higher risk of edema in the diabetes group is more likely due to the combination of insulin use in most diabetes patients. Some studies suggest that pioglitazone combined with insulin has a significantly higher probability of edema than pioglitazone alone (60). Although pioglitazone may be associated with water and sodium retention, it can also reduce the risk of myocardial infarction and ischemic stroke (61). A small sample size and a relatively short follow-up time may not reveal the entire spectrum of side effects; thus, the side effects of pioglitazone on NAFLD patients require a larger sample size and a longer follow-up time to get relatively true results.

The limitations of the article are related to the research design and the biochemical and histological parameters. In terms of the research design, the doses of pioglitazone medication varied among studies (15 (37), 30 (36, 38–40), and 45 mg/day (11, 12)), as well as the treatment courses (3 (37), 6 (11, 40), 12 (36, 39), 18 (12), and 24 months (38)). Some studies implemented strict diet (11, 12, 36, 37, 39) and exercise (36, 39, 40) regimens, while some did not provide any information about lifestyle (38). In addition, the inclusion criteria were also inconsistent among studies: some trials enrolled only type 2 diabetics (40), while others also included prediabetics. Some prediabetic NAFLD patients may be included in the non-diabetic NAFLD patients. The proportion of gender differences between diabetes and non-diabetes patients was relatively different. As for the explanation of biochemical parameters, some articles did not cover the research indicators, accompanied by inconsistent units of results, and some studies did not list an average of changes before and after treatment, resulting in insufficiently accurate results. Due to the limitation of the number of studies, we included NASH and NAFLD for analysis. The involvement of both NAFLD (37, 39, 40) and NASH in the present study (11, 12, 36, 38) also enhanced the heterogeneity of the research.

In conclusion, this systematic review suggests the same efficacy of pioglitazone in non-diabetic and diabetic NAFLD patients in alleviating histopathology, liver enzymes, and HOMA-IR and reducing blood lipids. Furthermore, it did not elicit extra adverse effects. Large sample sizes and well-designed RCTs are required to further confirm these conclusions.

## Data availability statement

The raw data supporting the conclusions of this article will be made available by the authors, without undue reservation.

## Author contributions

YJ, MX and GL designed the study. ZW and HD performed the research and carried out the statistical analysis. YR, YZ, CM and HC wrote the manuscript. JT, CX and ML read and checked the paper. All authors co ntributed to the article and approved the submitted version.

## Supplementary material

Supplementary Figure 1 Changes of liver function with pioglitazone **(A)** AST, **(B)** ALT.

Supplementary Figure 2 Changes in metabolism with Pioglitazone **(A)** HOMA-IR, **(B)** FBS.

Supplementary Figure 3 Changes in weight and BMI with pioglitazone **(A)** weight, **(B)** BMI.

Supplementary Figure 4 Adverse effects with pioglitazone.

Supplementary Figure 5 Sensitivity analysis in NAFLD patients with Diabete or Prediabetes based on **(A)** ALT, **(B)** FBS, **(C)** HDL, **(D)** HOMA-IR.
