# Supplementary figures and images for: Response to pioglitazone in non-alcoholic fatty liver disease patients with vs. without type 2 diabetes: A meta-analysis of randomized controlled trials
Source: Front Endocrinol (Lausanne). 2023 Mar 29;14:1111430. doi: 10.3389/fendo.2023.1111430 (PMC10091905; doi:10.3389/fendo.2023.1111430)

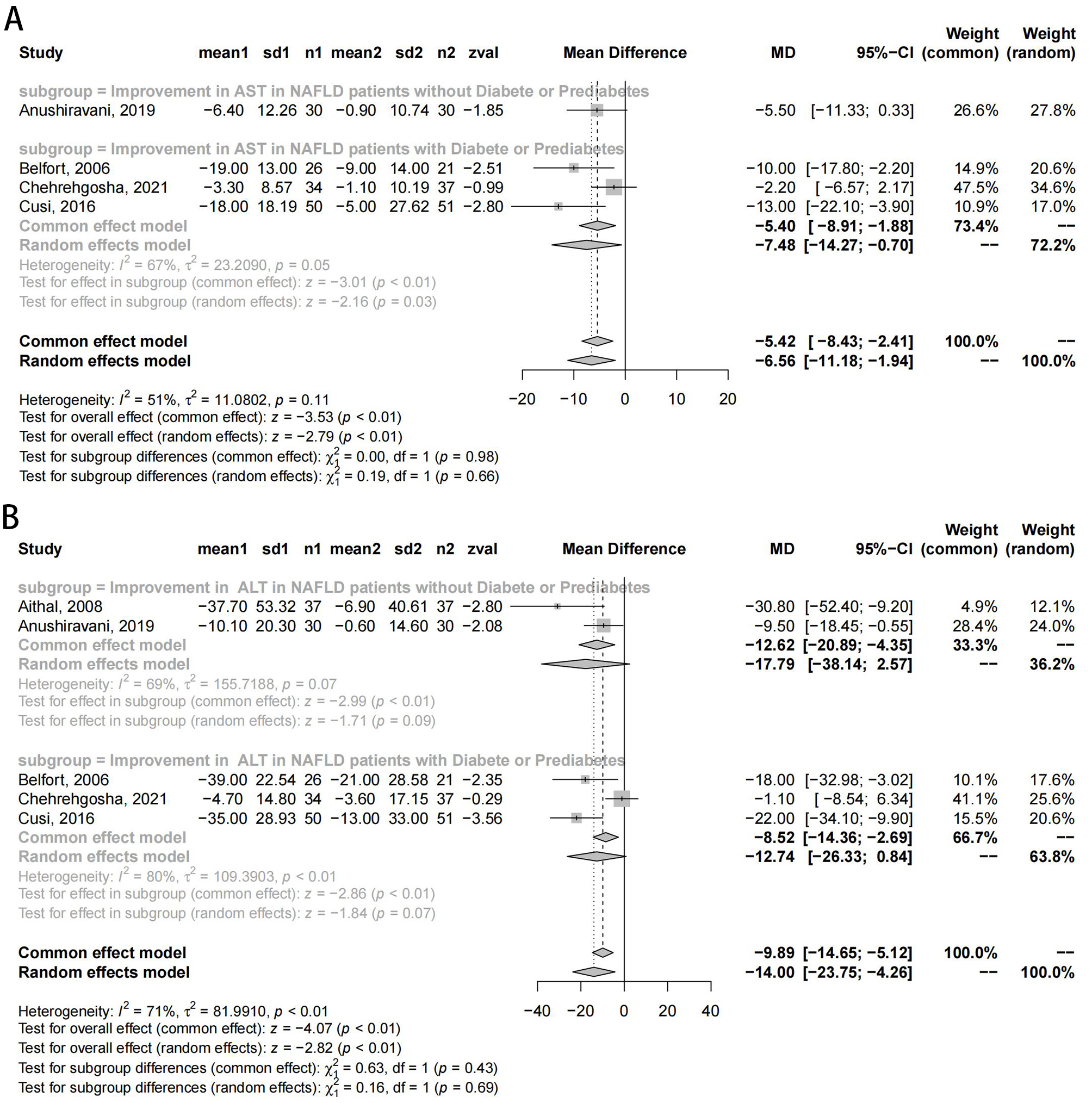

Supplement: Supplementary Figure 1 — Changes of liver function with pioglitazone (A) AST, (B) ALT. [file Image_1.jpeg]

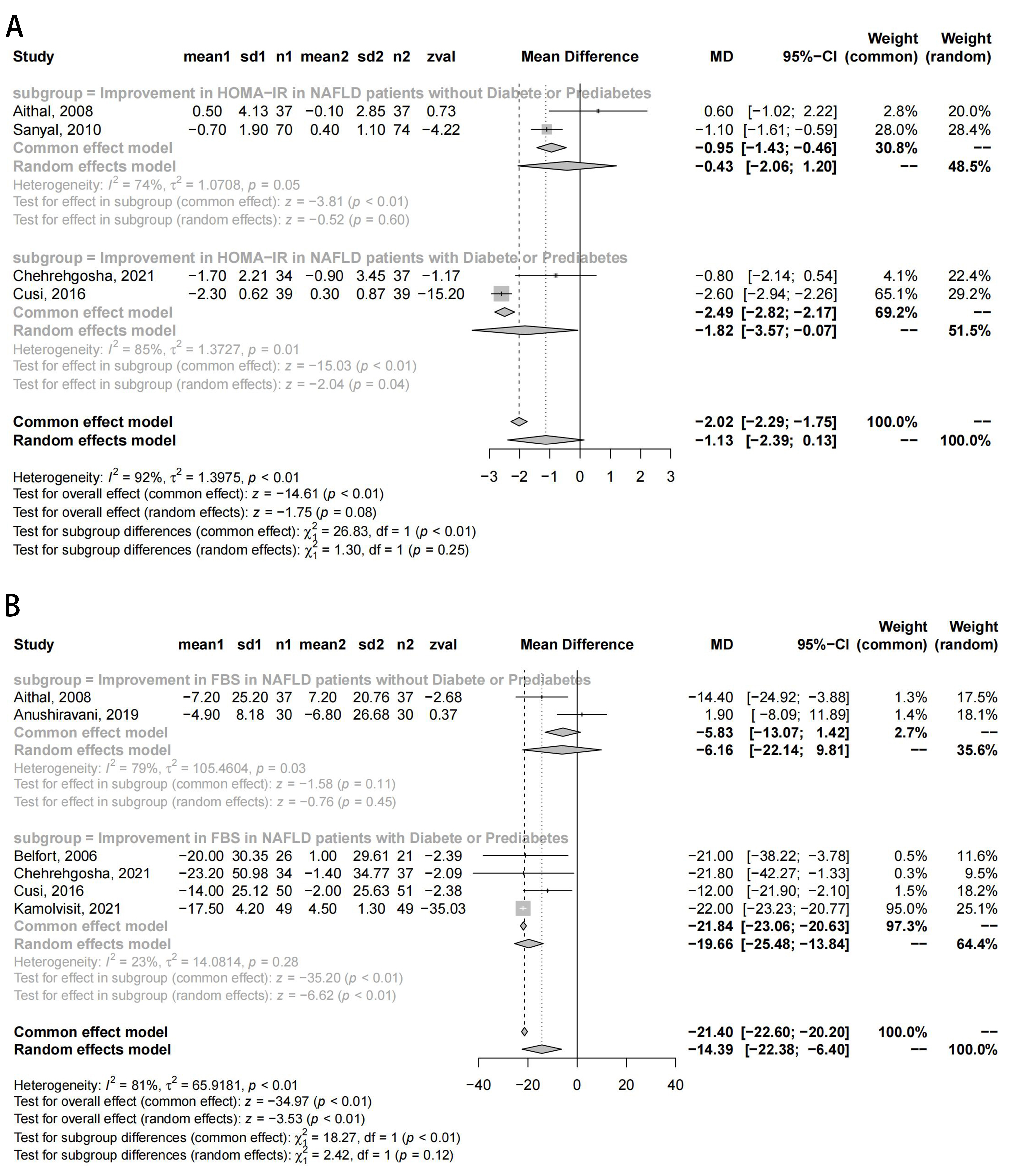

Supplement: Supplementary Figure 2 — Changes in metabolism with Pioglitazone (A) HOMA-IR, (B) FBS. [file Image_2.jpeg]

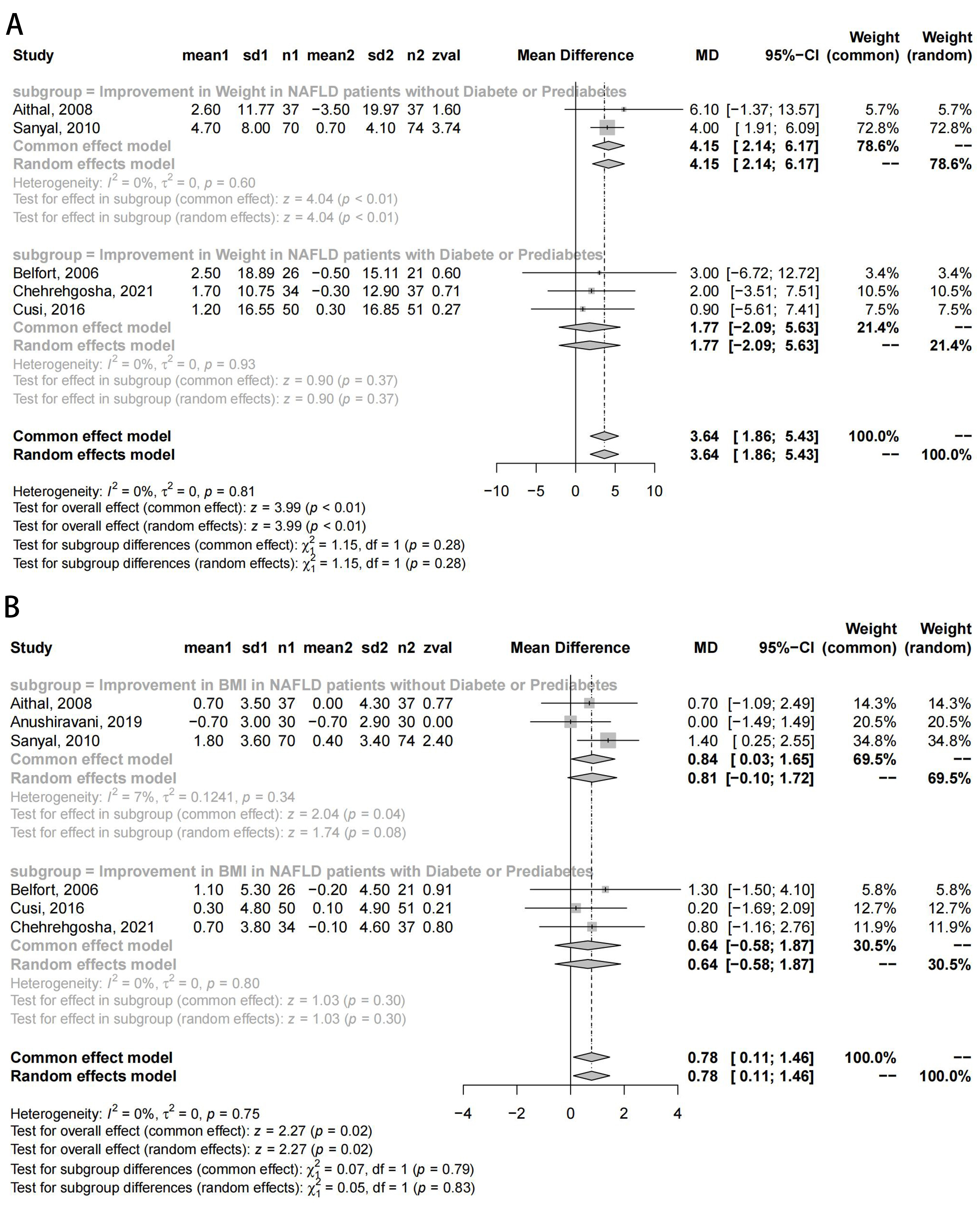

Supplement: Supplementary Figure 3 — Changes in weight and BMI with pioglitazone (A) weight, (B) BMI. [file Image_3.jpeg]

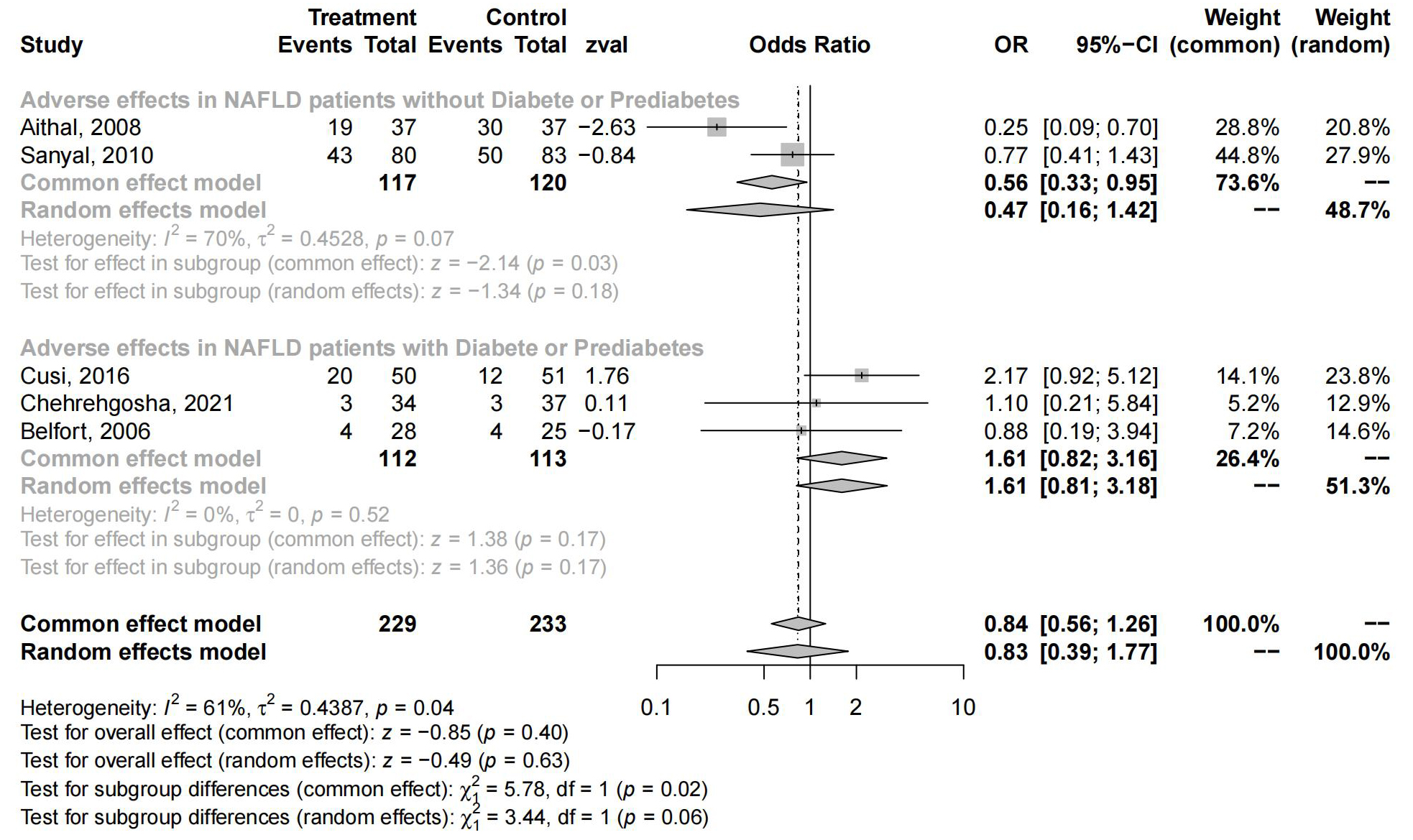

Supplement: Supplementary Figure 4 — Adverse effects with pioglitazone. [file Image_4.jpeg]

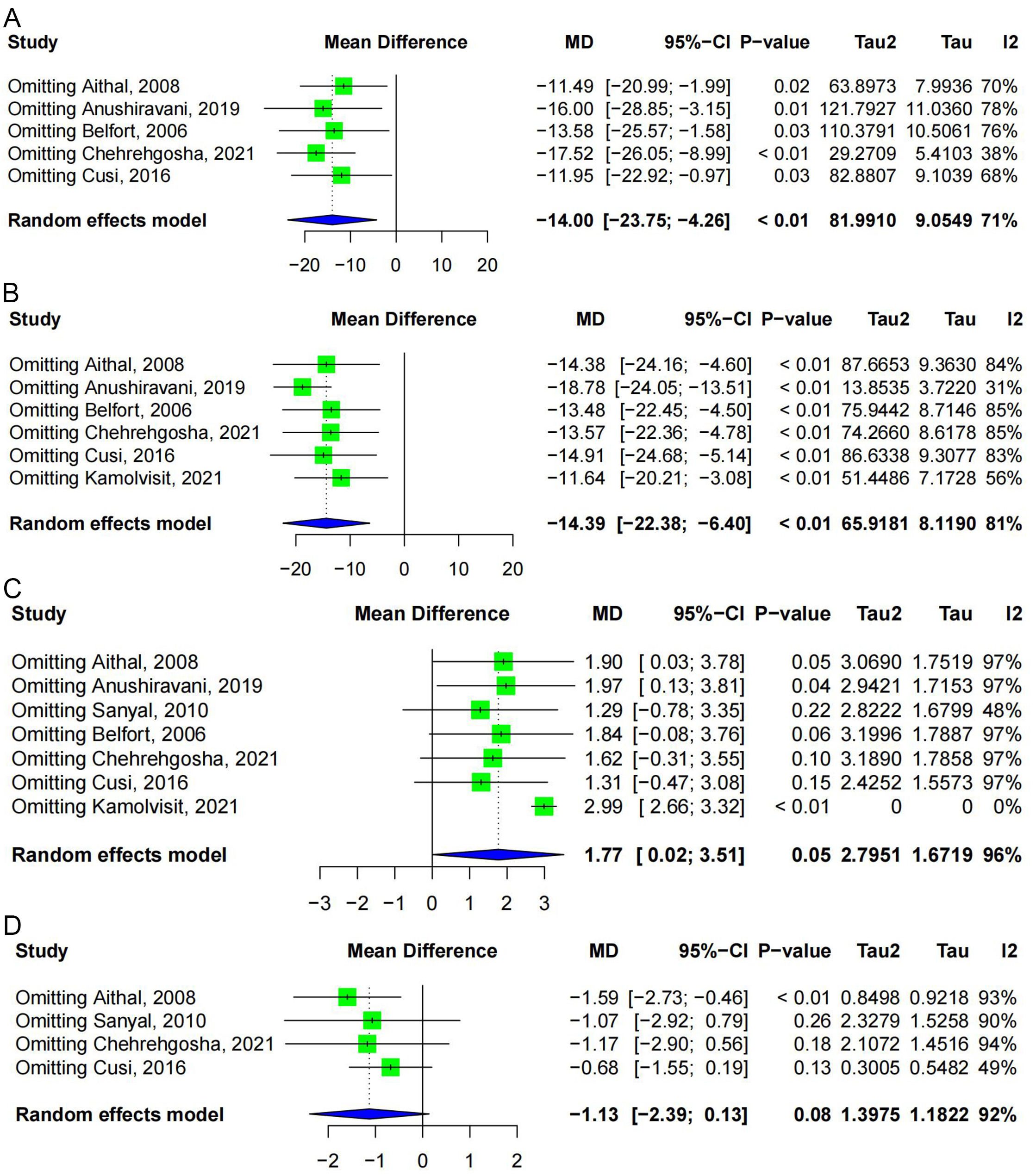

Supplement: Supplementary Figure 5 — Sensitivity analysis in NAFLD patients with Diabete or Prediabetes based on (A) ALT, (B) FBS, (C) HDL, (D) HOMA-IR. [file Image_5.jpeg]

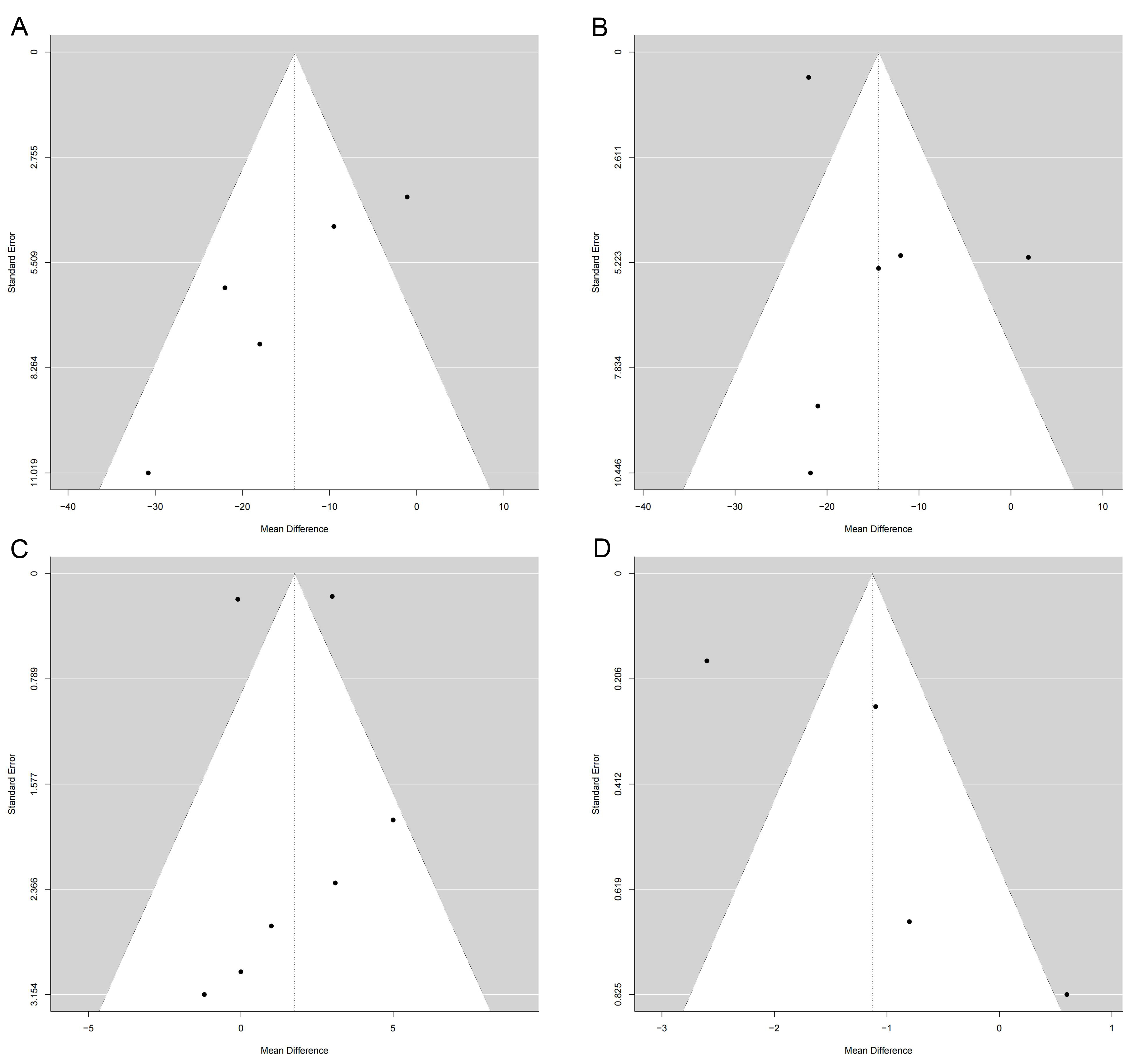

Supplement: Supplementary file 6 [file Image_6.jpeg]
