# Supplementary material for: Response to pioglitazone in non-alcoholic fatty liver disease patients with vs. without type 2 diabetes: A meta-analysis of randomized controlled trials
Source: Front Endocrinol (Lausanne). 2023 Mar 29;14:1111430. doi: 10.3389/fendo.2023.1111430 (PMC10091905; doi:10.3389/fendo.2023.1111430)
Supplement: Supplementary file 7 [file Table_1.docx]

| Variable | With Diabete or Prediabetes | | Without Diabete or Prediabetes | |
| --- | --- | --- | --- | --- |
|  | Pioglitazone | Placebo | Pioglitazone | Placebo |
| ALT (U/L) | -26.23 | -12.53 | -23.9 | -3.75 |
| AST (U/L) | -13.43 | -5.03 | -12.61 | -3.64 |
| Weight (kg) | 1.8 | -0.17 | 3.65 | -1.4 |
| BMI | 0.7 | -0.07 | 0.6 | -0.1 |
| HDL (mg/dL) | 3.75 | 1.5 | -0.13 | -0.73 |
| LDL (mg/dL) | -9.25 | -8.68 | -3.96 | -3.38 |
| HOMA-IR | -2 | -0.3 | -0.1 | 0.15 |
| FBS (mg/dl) | -18.68 | 0.53 | -6.05 | 0.2 |
| Cholesterol (mg/dl) | -9.68 | -8.48 | -5.69 | -8.11 |
| Triglyceride (mg/dl) | -53.5 | -6.53 | -18.75 | -16.63 |

**Supplementary Table 1. Mean Changes in different Features after Treatment for Baseline.**
